# A Dutch Study of Remarkable Recoveries After Prayer: How to Deal with Uncertainties of Explanation

**DOI:** 10.1007/s10943-023-01750-6

**Published:** 2023-02-04

**Authors:** Elena Bendien, Dirk J. Kruijthoff, Cornelis van der Kooi, Gerrit Glas, Tineke Abma

**Affiliations:** 1 Leyden Academy on Vitality and Ageing, Rijnsburgerweg 10, 2333 AA Leiden, The Netherlands; 2 Faculty of Theology, Vrije Universiteit (VU), Amsterdam, The Netherlands; 3 Faculty of Humanities, Vrije Universiteit (VU), Amsterdam, The Netherlands; 4 Leiden University Medical Centre, Leiden, The Netherlands; 5 Law, Ethics and Medical Humanities, Amsterdam University Medical Centre, Location VUmc, Amsterdam, The Netherlands

**Keywords:** Healing after prayer, Remarkable recoveries, Horizontal epistemology, Explanatory frameworks, Uncertainty

## Abstract

This article addresses cases of remarkable recoveries related to healing after prayer. We sought to investigate how people who experienced remarkable recoveries re-construct and give meaning to these experiences, and examine the role that epistemic frameworks available to them, play in this process. Basing ourselves on horizontal epistemology and using grounded theory, we conducted this qualitative empirical research in the Netherlands in 2016–2021. It draws on 14 in-depth interviews. These 14 cases were selected from a group of 27 cases, which were evaluated by a medical assessment team at the Amsterdam University Medical Centre. Each of the participants had experienced a remarkable recovery during or after prayer. The analysis of the interviews, which is based on the grounded theory approach, resulted in three overarching themes, placing possible explanations of the recoveries within (1) the medical discourse, (2) biographical discourse, and (3) a discourse of spiritual and religious transformation. Juxtaposition of these explanatory frameworks provides a way to understand better the transformative experience that underlies remarkable recoveries. Uncertainty regarding an explanation is a component of knowing and can facilitate a dialogue between various domains of knowledge.

## Introduction

Julia was diagnosed with post-traumatic dystrophy in 1990 (also known as CRPS) and became Dick's patient in 1992. She had pain in the right side of her body and was wheelchair bound. As a general medical practitioner (GP) Dick had a moderately large practice in a rural region of the Netherlands. His patients had various socio-economic backgrounds. He knew Julia’s medical history well. In 2007, after 17 years of suffering, Julia and her husband took part in a prayer healing session, that was organised by a well-known Dutch evangelist. After the prayer Julia stood up from her wheelchair and started walking around without a trace of pain. Her physical condition has remained stable during the past 15 years. Dick was pleased but also intrigued by Julia’s sudden full recovery. In search for an explanation he conducted a literature study but came up empty-handed. His inquiry led to research, supervised by an interdisciplinary team, consisting of a theologian, a psychiatrist-philosopher, a social scientist and a qualitative researcher in the field of medical humanities.

The turn to patient-centred medicine has been accompanied by an increased interest in the spiritual needs and beliefs of the patients (Mezzich, 2011; VanderWeele, 2017). This is reflected in publications about the influence of spirituality on wellbeing and other measures of quality of life. Some of these studies focus on healing after prayer (Banerjee et al., 2014; Miranda et al., 2019). With healing after prayer (further HP) we mean that a person’s health improved after intercessory, individual, or other types of prayer.

In Western countries HP was considered to be controversial as a field of medical and social research for a long time (Andrade & Radhakrishnan, 2009), but the number of publications that address the positive effects of prayer on health is steadily growing today (Shattuck & Muehlenbein, 2020). Most of the available empirical research on HP has been conducted with the use of Randomised Control Trials (RCTs) (Hodge, 2007) and usually reflects scepticism about the positive effect that prayer can have on a person’s health (Roberts et al., 2009). Only a handful of published empirical studies make use of qualitative methodologies (Austad et al., 2020; Harris & Koenig, 2016; Helming, 2011).

In RCTs, prayer is usually operationalised as an intervention, with a possible cause-effect (or even dose–effect) relationship between action and outcome. Some concerns about RCT as a suitable method to study HP are based on a large variety of HP-practices and the validity of operationalisation (Chibnall et al., 2001; Pagliaro et al., 2018). Can prayer be conceived as an act that is demarcated in time and can it be quantified in terms of frequency, strength, fervency, numbers of intercessors or who prays to whom (see e.g. Klitzman, 2022)? Besides, the outcome may extend beyond the usual, clinically measurable variables, and encompass changes in body, mind and spirit (Kruijthoff et al., 2021a, 2021b). In short, underneath the epistemological question how to study HP (De Aguiar et al., 2017), lies the conceptual issue how to understand a phenomenon that does not fit well with the currently dominant biomedical paradigm, that is based on the presumed duality between body and mind.

Against this background, we present a qualitative study based on the 14 (out of 27) cases, which were evaluated by a medical assessment team at the Amsterdam University Medical Centre, location VUmc (for review of the medical data of all 27 evaluations see Kruijthoff et al., 2022a). We will use the terms healing and recovery interchangeably. Recovery is understood as a long-lasting or permanent clinical improvement of the medical condition.

Each case we study has a well-documented medical history and has been submitted to a rigorous assessment by an independent medical team, concluding that the recovery could be medically remarkable or unexplained. Each case is characterised by an experience of (sudden) recovery related to prayer, and the participants are all inclined to search for other than medical explanations for the recovery. Our aim is to investigate how people who experienced remarkable recoveries, re-construct and give meaning to these experiences, and examine the role epistemic frameworks that are available to them play in this process. We demonstrate how established medical epistemologies are put to the test and how conflicting frameworks of understanding interact and are dealt with by the participants.

## Theoretical Perspective

The challenge of the choice for a theoretical framework that can help studying and interpreting HP cases from a multidisciplinary perspective, lies in the absence of developed theoretical approaches that match the existing data (Levin, 2020). Reports on remarkable recoveries range between cases that are (un-)related to HP but are medically verified (Engebretson et al., 2014), cases that are described on the Lourdes pilgrimage site (François et al., 2014) and self-reported narrative accounts of patients, to name just a few. The authors who endeavour to provide an explanation for HP, usually take an eclectic approach. Barasch (2008) makes an attempt to summarise the processes that can have influenced remarkable recoveries, such as psychosocial interventions (Spiegel et al., 2007), biological modifiers, diets, psychological states like mindfulness or meditation (Rediger & Summers, 2007), immune responses and social connections. He also points to the lack of thorough accounts of the cases, and the difficulty to replicate the conditions under which these recoveries took place (see also Rediger, 2021). These accounts indicate that biomedical, biopsychosocial and even holistic explanatory frameworks can be of use when addressing certain physiological, lifestyle and relational aspects of remarkable recoveries (see, e.g. Engebretson et al., 2014 about social connections), but they come short in describing the spiritual or the transformative experiences of the patients.

Communication about remarkable recovery is a challenge of its own. Cases of medically unexplained symptoms (MUS) can be instructive about how this kind of communication unfolds. The interaction between patients and doctors is crucial in situations where diagnosis, treatment and recovery prospects do not fit within the mainstream clinical practice. It can result in the patient being treated as an “unreliable narrator of bodily events” (Scarry, 1987). In the absence of an evidence-based explanation for the symptoms of a disease or a sudden recovery, the patient-doctor interaction can be characterised by conflicting feelings of uncertainty or hope on the part of the patient, and mistrust or even animosity on the part of the doctor (Greco, 2012, 2017).

The positions that patients and doctors find themselves in, can affect the credibility of both parties. Safe ways out to explain a remarkable recovery from the point of view of the specialist, are to admit that the patient was misdiagnosed, or to describe the condition as self-resolving, or to suggest that the recovery is nonreplicable (Barasch, 2008). The patient can feel torn between relief and fear that the recovery is only temporary, and not knowing what to further expect from the doctor. The doctor can become nervous and start second-guessing the diagnosis that was made in the first place (Salmon et al., 2007). In such cases the consultation can turn into a battle (Wileman et al., 2002) about whether the recovery has actually taken place or, broader still, about the legitimacy of the parties to ascertain the improvement.

The examples with remarkable recoveries and MUS demonstrate the same shortcomings where explanation and communication are concerned. The theoretical approaches and communicative tools that are used to interpret and discuss these cases, do not address the spiritual aspects of healing, even though the positive influence of spirituality on physical health is well-established (Koenig, 2015; Thoresen & Harris, 2002).

Our conceptual framework is built on a combination of approaches: positive health, horizontal epistemology, which addresses amongst others the asymmetry in the doctor-patient interaction, and trans-somatic recovery, that allows to place the recovery in the context of the person’s spiritual development.

The framework of positive health focuses on agency and the adaptability of the patient, who may still be able to live a good life, after having been diagnosed with a chronic condition. Recovery is defined as ‘the ability to adapt and self-manage in the face of social, physical and emotional challenges’ (Huber et al., 2011, p 343). This definition reaches beyond the healthcare system, since it includes non-health factors (Andersen & Knudsen, 2015), such as life-events, identity-forming and sense-making. Attention to self-management in the face of the challenges that a positive health-framework promotes, allows us to link our study to the field of research on biographical disruption and identity (Bury, 1982; Charmaz, 1983, 1995). Our attention will however be less on the biographical disruption as a consequence of a medical diagnosis, and more on the disruption resulting from spontaneous recovery followed by restoration of the self (Locock & Ziebland, 2015).

Horizontal epistemology (Abma, 2020) refers to the way of knowing where a hierarchic division between various types of knowledge (scientific, expert, experiential) becomes restrictive. Fricker (2007) has pointed to the epistemic injustice of hierarchic systems of knowledge, where certain people are being systematically wronged in their capacity as knowers and denied the possibility to tell their story (Carel & Kidd, 2014). When certain perspectives and types of knowledge are structurally left out of the process of knowledge production, this will lead to a limited understanding of our world.

Horizontal epistemology suggests that different epistemic perspectives and different types of knowledge should be dealt with as equally important in the interpretation of research findings. The approach has two advantages: it includes experiential knowledge as a legitimate source of knowing (Sturmberg & Martin, 2008) and it allows for a broad dialogue between various types of knowledge and knowing, including tacit forms of knowledge (Polanyi, 1958).

Horizontal epistemology is performative by nature; it is enacted in the interaction between various discourses about illness and recovery. It generates new insights by bringing various disciplines and stakeholder perspectives together, based on empirical data. On a positive side, it is transformative to our understanding of complex phenomena, and it broadens the existing explanatory possibilities of complex cases. On a challenging side, horizontal epistemology is rooted in interpretation of data, which includes interpretation by the researcher, who uses personal experiences as a source of knowledge and explanation. Here the role of the researcher is not that of a distanced impartial investigator. That is why ethical and emotional aspects of knowing carry a heavy weight within horizontal epistemology (Abma, 2020).

Horizontal epistemology entails the possibility that the medical specialist is no longer the (only) person who decides whether recovery has taken place. In fact, the self-reported functionality of a patient can outweigh the available medical readings (Kruijthoff et al., 2021b). This leads to a broader issue, whether recovery can be understood on the basis of untraditional somatic explanations. There is a wealth of critical literature about how patients and doctors use somatisation in order to explain a condition that is not supported by the available medical measurements (Greco, 2017; Salmon, 2006). Following this logic, in order to be legitimate, recovery should be substantiated by quantitative somatic measurements or by standardised verbal reports of the patient’s experience. Recoveries that cannot be measured or articulated by standardised means, represent a challenge to explanation.

To do justice to this complexity, we frame our findings in terms of trans-somatic recovery. With this modifier we aim to highlight dimensions of recovery that go beyond its customary physical and mental characteristics. The term draws attention to the transformative and transcending nature of the recovery experiences.

Transformative recovery refers to healing experiences that extend beyond the functionality of body and mind. The term refers to instances in which healing leads to existential self-reflection, spiritual development, and/or religious transformation. Trans indicates the transcending aspect of recovery, understood as a process that brings patients to a level at which they can see their existence from a new overarching perspective. The transformative experience places their existence in a different light. The new perspective does not erase or replace other experiential dimensions. It includes them and transforms them into a new, meaningful, but sometimes disruptive, experience. Such experiences can vary from physical sensations, mood changes, experiences of improved health, to feelings of belonging to the universe or of undergoing a radical change, for example due to an encounter with God (Austad et al., 2020; Lundmark, 2010). All these experiences can unfold simultaneously and influence one another.

All in all, trans-somatic recovery does not imply that the body has become non-essential or subsidiary to other aspects of life. It rather means that there exists no hierarchy between the various dimensions of recovery, including the spiritual dimension, and that we should focus on discourses that do justice to the inclusive nature of transformative and transcending healing experiences.

## Methods

The findings presented in this article form a part of the second author’s PhD study. The full design protocol of that study has been published elsewhere (Kruijthoff et al., 2017). It is defined as a retrospective naturalistic case-based study (Abma & Stake, 2014) and consists of a preparation phase, that includes data collection, followed by three phases of analysis: medical assessment, qualitative data analysis and interdisciplinary meta-analysis. Most of the results of the PhD study have been published already (Kruijthoff et al., 2017, 2021a, 2021b, 2022a, 2022b). For the PhD study two sets of data have been used: medical records of (former) patients and transcripts of qualitative interviews with the patients. In this article we use the second set of data and focus on qualitative data analysis of the 14 interviews.

### Procedure and Participants

In 2016 a Dutch newspaper announced that the Faculty of Theology of the Vrije Universiteit Amsterdam would, in cooperation with researchers from the Amsterdam University Medical Centre, location Vumc, be supervising a PhD study, which was being conducted by a GP, on the topic of healing after Christian prayer. The article generated a huge response, both positive and critical. In due course the second author received 83 reports from prospective respondents with accounts about their HP (for the detailed overview of all cases and the follow-up data that was collected in 2019 and 2021 see Kruijthoff et al., 2022a, 2022b).

The research protocol (Kruijthoff et al., 2017) describes in detail the criteria for inclusion in the study: the participants must have a well-documented medical history, followed by subsequent recovery related to Christian prayer. Based on these criteria 27 cases were presented for review to a medical assessment team, consisting of five medical specialists in the fields of internal medicine, haematology, surgery, psychiatry, and neurosurgery. According to the research protocol they represent a variety of ideological backgrounds, both agnostic and religious, in order to minimise bias (Kruijthoff et al., 2017). None of them consider HP as a medical intervention. All available medical files were collected – with written informed consent of the participants – from the medical institutions and hospitals where they had been treated (for the detailed overview of the 27 cases see Kruijthoff et al., 2022a).

The medical assessment team marked 14 (out of 27) cases as possibly medically remarkable or unexplained, and selected them for in-depth interviews. The term ‘medically remarkable’ refers to a healing which is surprising and unexpected in the light of current clinical and medical knowledge and that has a remarkable (temporal) relationship with prayer, while ‘medically unexplained’ indicates that no scientific explanation could be found at the time of assessment (Kruijthoff et al., 2017).

Subsequently the first author conducted semi-structured interviews with the 14 participants in 2017–2019. The transcripts of the interviews form the primary data for this article. For the participants’ characteristics see Table 1.

**Table 1:** Participant demographic characteristics

	Sex	Age category at the moment of healing	Education level^*^	Religious affiliation at moment of healing	Time interval between interview and healing(years)	Illness
1	M	65–70	Low	Evangelical	1	Partial spastic hemiparesis after Cerebro Vascular Accident (CVA)
2	F	40–45	High	Reformed protestant	11	Pelvic instability and Hearing impairment
3	F	35–40	High	Pentecostal	11	Crohn’s disease
4	F	60–65	High	Baptist	3	Multimorbidity**
5	F	50–55	High	Reformed protestant	9	Multiple sclerosis(Disability score EDSS 6,5/10)
6	F	25–30	High	Non-religious	2	Anorexia nervosa
7	M	45–50	Medium	Reformed protestant	14	Iatrogenic aorta dissection
8	F	50–55	Medium	Baptist	6	Parkinson’s disease
9	F	30–35	High	Religious without church affiliation	4	Ulcerative colitis and Psoriasis with artritis
10	F	30–35	Medium	Reformed protestant	11	Ulcerative colitis, about to undergo colectomy
11	M	50–55	Medium	Reformed protestant	4	Drug-induced hepatitis with impending liver failure
12	M	50–55	Medium	Religious without church affiliation	8	Cuff rupture shoulder
13	F	35–40	High	Reformed protestant	6	Congenital hearing impairment
14	M	50–55	Medium	Reformed protestant	16 and 13 respectively(2 separate healings after prayer)	Alcohol addiction; One-sided posttraumatic dystrophy and nerve entrapment in leg

*Low: primary school only

Medium: primary school and medium secondary education

High: primary school and high secondary education/university

**Astma, disability due to inflammatory osteo-artritis, impaired hearing, incontinence

The participants are women (*N* = 9) and men (*N* = 5), between 29 and 71 years old. They are all white Dutch (*N* = 13) and Belgian (*N* = 1) citizens. The duration of their medical conditions, prior to their healing, varies between 7 weeks and 30 years. The period between the healing and the interview varies between one and 16 years, on average 8 years. The medical conditions from which they experienced recovery are: cuff rupture of the shoulder, pelvic instability and one-sided deafness, Crohn’s disease, cerebrovascular accident (CVA), iatrogenic aortic dissection, ulcerative colitis (*N* = 2) and psoriatic arthritis, multiple sclerosis (MS), anorexia nervosa, Parkinson’s disease, drug-induced hepatitis, severe asthma and impaired hearing, alcohol addiction and posttraumatic dystrophy, and congenital hearing impairment. An analysis of these cases has been published elsewhere (Kruijthoff et al., 2022a, 2022b). Some of the cases were analysed more extensively in detailed case studies (Kruijthoff et al., 2021a, 2021b).

The interview guide included: general background information, social and physical conditions during childhood, (professional) education, religious background, marital status, employment, history of the illnesses, symptoms before and after the recovery, a detailed reconstruction of the moment/period of recovery, including bodily sensations, the respondent’s knowledge about HP prior to recovery, the time frame between the prayer and the experience of being healed, the reactions that the participants received to the recovery, the impact of the recovery on the participants’ lives and the meaning they ascribe to the recovery.

The interviews were conducted at the homes of the participants (*N* = 13) and at the university (*N* = 1). The duration of the interviews was 1, 5—2 h; they were audio-recorded and transcribed verbatim. The final versions of the interviews were adjusted in accordance with the suggestions of the participants during a member check. Subsequently the interviews were presented to the medical assessment team for final evaluation.

### Data Analysis

The first and the second author conducted the analysis of the interviews, which was inspired by the principles of constructivist grounded theory (Charmaz, 2008, 2014). Use was made of ATLAS.ti software for open and focused coding. An iterative approach to the data collection and analysis has been applied. The insights obtained from the analysis of the first interviews and the feedback provided by the interviewer regarding non-verbal interaction, were discussed and incorporated in the later interviews. Hence, the question about personal sense-making in relation to the healing experience was posed more explicitly in the later interviews.

The main guideline during the open coding was interacting with the data and comparing the codes from different interviews that were generated by the two authors. In order to avoid bias, in vivo codes were prioritised. The research goal, namely to look for categories that would contribute to an exchange between various explanatory frameworks and allow for juxtaposition, guided the researchers during the process of comparing codes and notes. During the focused coding we intentionally searched for categories that could enrich or transcend monodisciplinary discourses, in order to match the complexity of the data and to allow for an elaborated epistemological framework to emerge.

We started theoretical sampling by comparing our data with the medical explanatory framework that was available to our participants and to our research team (through participation of the medical assessment team). In search for theoretical saturation and led by the rich data at hand we eventually broadened our theoretical sampling by investigating whether the life-course, spiritual-quest and sense-making explanatory frameworks might answer our question as well. At that stage of analysis we used not only inductive but abductive logic of reasoning as well (Reichertz, 2019), which allowed for a better understanding of surprising findings (e.g. similar physical experiences by various participants) and emergent themes, like the role of miracles in the life of our participants, which ‘invoked imaginative interpretations’ among the members of the research team (Charmaz, 2008, p 157)). Our analysis pointed out to a juxtaposition of three explanatory frameworks: medical, life-course and religious and spiritual transformation.

### Reflexivity

This study was initiated because of a personal experience and curiosity of one of the authors. Constructivist grounded theory does not demand from the researcher to be totally impartial during the research process, but rather to continuously reflect on how the researcher’s perspectives and also the context within which research takes place, can be made explicit (Charmaz, 2008). Such reflexivity is in accordance with the demands of horizontal epistemology as well. To ensure that personal perspectives of the researchers do not determine the results of the analysis, the research team had regular meetings in the course of several years, during which they reflected on the results, the process of the research and their own role in it. The second author has remained in contact with the participants to date and informs them regularly about the progress of the research. One of the participants became a co-author of one of the published articles (Kruijthoff et al., 2021a).

## Findings

A noteworthy feature of the interviews is the temporal correlation between the moment of prayer and the experience of healing. In 10 cases the actual healing was experienced instantaneously, and in four cases the onset of the healing started immediately after the prayer and then continued for several days or weeks. Most of the participants did not have any previous experience or detailed knowledge about HP. Those who attended a service (*N* = 8) had low or no expectations. The participants who prayed on their own (*N* = 6) asked for an end to their sufferings one way or another.

**Fig. 1:**
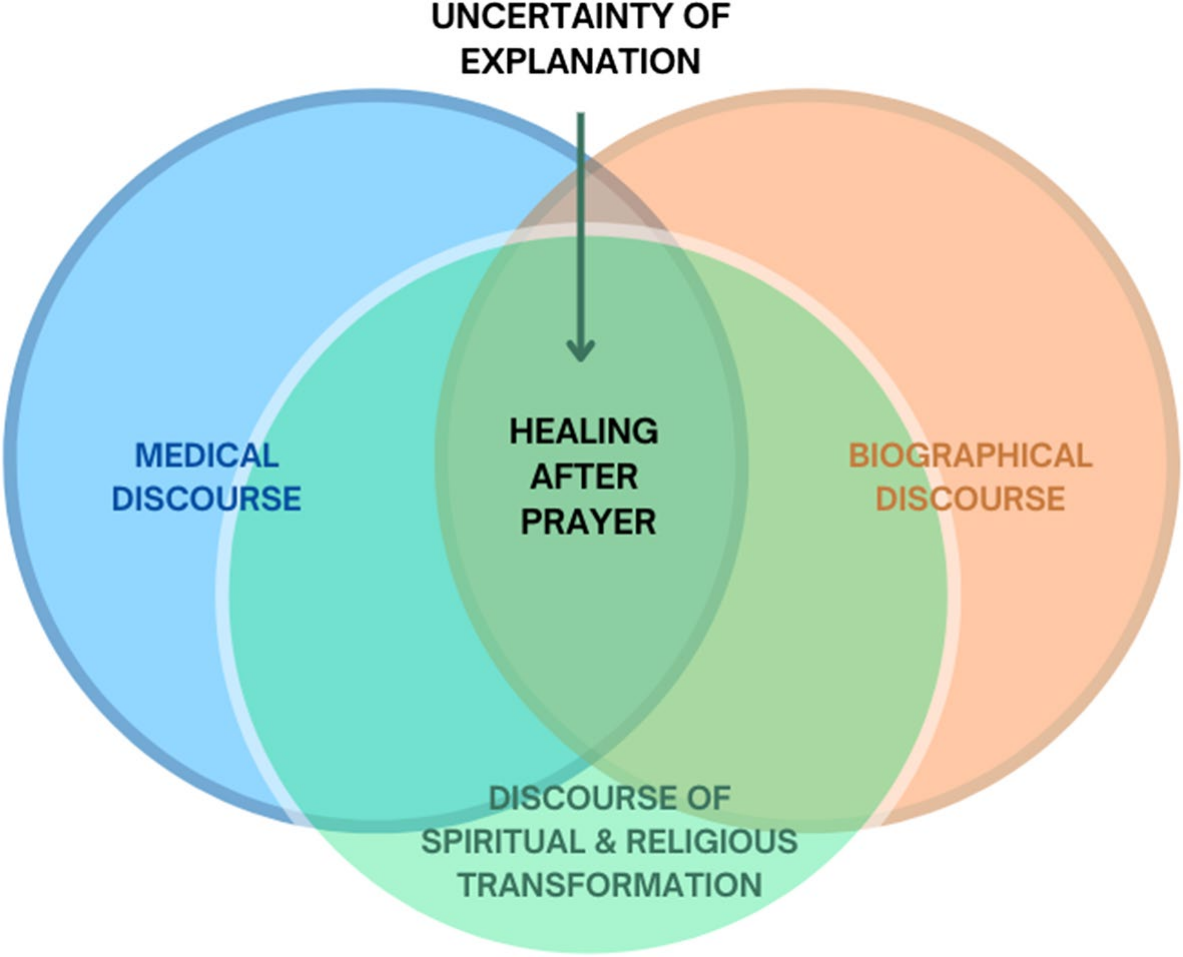
Key finding

Each story about a remarkable recovery emerges from a combination of different discourses, which we summarised in three themes: ‘authenticity of the illness and recovery (un-)warranted by medical discourse’; “remarkable recoveries in the context of biographical discourse”; and “feeling healed and whole again: discourse of spiritual and religious transformation”. For schematic representation of the findings (see Fig. 1).

### Authenticity of the Illness and Recovery (un-)Warranted by Medical Discourse

The first theme is about the role of the medical discourse and the certainty of explanations during the interaction between medical specialists and participants, from the perspective of the participants. They talk about a large part of their illness and recovery in clinical terms, using a biomedical explanatory framework. Although they are convinced that their recovery is associated with the influence of a divine source, each of them seeks medical confirmation for the authenticity of their condition and recovery. Each case starts with a history of the disease, hence large parts of the interviews contain meticulous descriptions of diagnoses and impairments, as experienced by the participants:

I had osteoarthritis, abdominal pain, depression, I took 22 pills in the end, 60 mg morphine, prednisone. I had to be washed twice a week. Then …they scheduled CT-scans, bronchoscopy, breathing tests, blood tests. And I had braces on my hands and on my knee. Then I got a device at home with flasks of oxygen and medicines, and I had to put a tube into my mouth and then go to sleep. (P4) (participant four, see table 1)

The use of medical terminology is abundant and appears to give more strength to the accounts of the participants, in order for their suffering to be acknowledged:

I ended up in hospital with a hernia at L5-S1. And then there was a rheumatologist standing next to my bed, and they said, you have Bechterew and you will never recover. Later they reversed that diagnosis, but they said that my pelvis was totally broken… (P2)

A noteworthy aspect of the last quote is the definitiveness with which, according to the participant, the medical specialist communicates the diagnosis, which is similar to experiences of other interviewees. For some of them this leads to taking decisions that worsen their condition. The participant with Crohn’s disease hears that her condition is incurable when she is 24. Her reaction can be described in terms of diagnostic shock (Belgrave & Charmaz, 2015). As she puts it, she feels devastated, because she has other plans for her life. Her distress and unwillingness to accept the diagnosis makes her look for alternative treatment. She stops with the prescribed medication and embarks on a diet, which makes her condition worse. Looking back, she calls it a big mistake, but she emphasises that the certainty with which the label ‘incurable’ was given, was not helpful either.

A message about a chronic condition that is delivered unemphatically can cause, to use Hadler’s metaphor, an erosion of dreams (1996) and stimulate a rebellious response, as with a participant who has a severe hearing impairment:

In the hospital I was told: you cannot choose a social profession, because your hearing is severely impaired. I was 11. And I was such a social being! That clashed completely with who I was. So, I became defensive. I did not want to be deaf. And I wanted to stop not-wanting-to-be-deaf. (P13)

This participant chooses for a profession for which interactive skills are indispensable, but soon she has to stop due to a burnout. The ways in which our participants make use of- and react to the medical discourse, demonstrate their dependence on it and at the same time their wish to regain control of their lives.

According to the participants, the reactions of the medical professionals to the announcements about HP vary from incredulity, anger and irritation to neutral contemplation or sincere curiosity. The participants expect joy from the medical professionals, but the majority is confronted with doubt:

I had no complaints at all. [The doctors] didn’t know what to say. I sat there and thought: if I were talking to a patient who says to me that he is feeling well, I would reply ‘how nice and how did that happen?’. And now it was like ‘we think that is very odd’. It became even crazier when the doctor suggested ‘using maintenance medication.’ (P9)

The participant who had recovered from a cuff rupture of the shoulder just a few days before the scheduled operation, describes eloquently the reaction of his doctor:

I think of waltzing over to that hospital, but …the specialist was super sceptical when I said that the operation was no longer necessary. He became furious and started throwing Latin names at me. ‘Surely that muscle has not seen the Light!’ He didn’t give in, and I was not allowed to have an ultrasound, because ‘that muscle could not be healed anymore’. (P12)

The participant with CVA had a similar experience. His physiotherapist puts him through extra heavy physical tests on the walking belt, because, according to the participant’s account, he does not believe in his recovery. This participant mentions how angry the physiotherapist becomes and his own determination to prove his recovery: ‘I’d rather drop dead than stop.’

At least half of the interviews contain this kind of examples. Based on them, we suggest that medical professionals regard it as a challenge to think beyond the scope of their clinical experience and the biomedical concept of the disease. In several cases, however, the professionals do accept that contemporary medicine cannot explain each and every clinical phenomenon. The recovery from ulcerative colitis of one of the patients was confirmed by a medical examination. According to the participant, the specialist who conducted the test was astounded by the improvement. Another doctor formulates it explicitly: ‘Medically speaking I have to admit that something happened that I cannot explain. I cannot substantiate it, but this is what I see’.

Both participants and doctors keep looking for confirmation of the initial diagnosis and the recovery by using the medical explanatory framework, albeit for different reasons. The account of the participant recovered from MS is very telling:

I used to go to the neurologist in a wheelchair, but that time we went on the motorbike. That was such a kick! I wanted a new MRI. …Then [the doctor] called and said: the MRI is unchanged; we will not retract the diagnosis. That was very important to my story. On the one hand, I thought it was a real shame, because I would have so much liked to have all those spots gone. That would have been visible, tangible evidence for me. On the other hand, I can function normally, so it doesn’t bother me anymore. The only strange thing is that the neurologist never sent a letter to my GP. (P8)

This example shows how various epistemic frameworks can juxtapose, while both the participant and the doctor are searching for an explanation of the recovery. A somatic examination can increase the trustworthiness of the participant’s story, but it can also raise doubts about the correctness of the initial diagnosis. An unchanged MRI can lead to various conclusions: for the participant it is an additional proof of divine interference, for the medical specialist it entails the question of responsibility for the patient, who declares she is healed, whereas evidence tells otherwise. The possibility that the neurologist never sent a letter to the participant’s GP can be seen as a sign of uncertainty, time pressure or simply as a lack of communicative skills in cases of medical uncertainty.

A few participants with measurable improvements, receive acknowledgment of their remarkable recovery. One of the doctors asked the participant for permission to follow the process of his remarkable recovery. Another doctor shared the participant’s line of thought:

He says: ‘I have no explanation for it, I know one thing: we, doctors, really don’t know everything’. I asked: ‘What will you write down in your file?’ And he wrote ‘a spectacular improvement after prayer.’ (P5)

The medical staff seem to remain ambiguous about joining the celebration of their patients’ unexpected recoveries. According to one participant, her doctor put it as follows: ‘To be a doctor is not just to master the craft of treatment, it is about the art of healing. This is not an easy profession, and the theory does not always show you the way forward.’ In addition, the fact that recovery occurs after a prayer, makes all parties uncertain about how to articulate it.

### Remarkable Recoveries in the Context of Biographical Discourse

The second theme allows to look at the medical history of the participants from the perspectives of their life-course and spiritual development. Each of the interviews includes a life-story, where the recovery is placed into the social and cultural contexts of the participant’s life and their relationships with others. It also contains an account of the participant’s spiritual journey, including a detailed description of their experiences with HP. An in-depth analysis of one of the cases has been published elsewhere (Kruijthoff et al., 2021a).

Illness catches up with the participants at different stages in life and is often followed by a biographical disruption and changes in perception of self (Bury, 1982). In that respect their experience is not different from that of any other patient with a diagnosis of a chronic illness (Charmaz, 2000). Their life expectations come into conflict with the consequences of their debilitating condition. Therefore, some of them try to conceal their illness or to cope with its consequences, since they are unwilling to accept the label of for ever being a patient with a chronic condition (Hadler, 1996). The participant with MS diagnosis states bluntly: ‘The moment you tell them, you will become Multiple Sclerosis’ (P5).

The duration of the participants’ impairments varies between months and decades. The coping strategies are often directed at preservation of the participants’ psychological wellbeing. The participant with MS makes ‘armed peace’ with her illness, because she does not want to feel like a victim. The participant with a hearing impairment learns to hide her condition by lipreading so well, that people simply don’t believe her when she finally reveals it. The participant with an inflammatory bowel disease convinces herself that she is meant to accumulate all the hereditary conditions of her family, thus allowing the others to remain healthy, because she is the one ‘who can bear them best of all’ (P9). The ability to be self-reflective is used as a coping strategy as well. The participants tell openly that coping with their condition takes its toll on their psychological wellbeing, their relationships with others and their self-image. Some of them have severe psychological complaints as well, like suicidal thoughts (P2), depression (P9), burnout (P13) or various forms of psychosis or addiction (P6, P14).

Each participant has had a connection with Christian religion since childhood, but only a few of them speak about having faith in their early years. Two of them bring up a faith-versus-autonomy issue, namely how making your own choices can coexist with faith. One of them remembers seeing God as ‘dangerous’, because people make themselves dependent on God and therefore cannot live their own lives. Another participant does not believe in God because as a child she found it ‘too easy’ to make yourself dependent on such a force, which can turn you into a weak person. There are two patterns that unite all the accounts: at some point in their lives the participants embark on a quest for ‘their’ God, who would satisfy their spiritual needs. They also keep their relationship with God separate from the church as an institution, resorting to a privatised form of religion: ‘In church, I noticed, faith is something distant that you are told about, while for me it is something very personal’. (P9).

Some of the incentives to search for faith or to become converted, are feelings of loneliness, weak family ties, or previous experience with remarkable recovery. Several of the participants undergo changes in their faith, from unquestioning faith to faith that they call ‘relationships with God’, that meets their need to belong, to becoming part of a community. One of the participants explains it to a stranger as follows:

I believe very simply in God, as a child. I have a place where I can cry out, vent my frustration, share my joy. God gives me strength, he protects me. The man had to laugh, and I asked: ‘Do you have anything better?’ He had to think, and then said that, in fact, he didn’t. I said I’ll stand my ground then. (P1)

Another participant states directly: ‘We simply need each other. Some people do that in church, and that’s fine.’ (P6).

Four of the participants connect their faith with the witnessing of miracles. They do not use the term ‘miracle’ as a technical theological notion. They refer to miracle as an unexplained positive event, something transcending the rational world they are living in, an opening into a spiritual dimension. One of them witnesses the remarkable survival of a family member after a car accident. When a passer-by prays for the victim she comes back to life, after which our respondent embarks on a quest for his ‘relationship with God’. A participant who has recovered from anorexia nervosa, considers her own recovery to be a miracle and although she is critical about the church as an institution, she starts believing in God after that. In fact, all participants consider their recovery to be a gift of God.

The medical, life-story and spiritual-quest discourses come together in a description of the moment when the healing takes place, which is central in all interviews. Initially none of them sees a connection between the possibility to recover and faith. When the recovery takes place, it comes unexpected and can therefore not be interpreted as a result of high expectations. The attention to the somatic symptoms that the participants provide in the description of their medical condition, stands in contrast with the description of the prayer-moment and its consequences, whereby the physical sensations form only a part of the entire healing experience. Although each of the 14 healings is experienced differently, the discourse that the participants use to describe them, can be called poetic. It is affective, full of metaphors and often refers to the sensation of being freed from something malignant:

I have wondered many times, what is trapped inside me? And when they prayed for me, someone put his hands on my back. Later I felt as if my back was completely bruised, as if someone had drawn two claws from it. It felt like something had been ripped out. Later I thought, apparently there was something that I was suppressing with medication, but that is no longer there. Yeah, it sounds a bit crazy… (P9)

The participants tell us about the affective side of the healing, that was experienced as being ‘touched inside your head and feeling a slow current going from your toes through the entire body’ (P2), ‘a sudden feeling of joy and the warmth of a hand felt on the exact place’ where the aorta was damaged (P7), the feeling of quiet and such a profound peace within, ‘as if somebody had wrapped a blanket around me and I felt that I am allowed to be’ (P4), ‘a large warm cloud, and the feeling that something is happening now, as if a small net has been taken away from my brain’ (P8). The last quote belongs to the participant with Parkinson, who adds: ‘It seems as if God has operated on my head’, an interesting addition that can be seen as an attempt to reconcile the medical and spiritual discourses from an overarching, transcendent perspective.

### Feeling Healed and Whole Again: Discourse of Spiritual and Religious Transformation

The third theme addresses the transformative power of healing: the changes in self-image of the participants before and after their recovery and the meaning that they give to the healing. The transformation of the self-image can undergo gradual as well as abrupt changes from the period before the diagnosis, during the disease, which is characterised by a partial loss of self, and after the recovery, resulting in a restored self (Charmaz, 2000). The onset of the disease shows how personality features become somatised, i.e. dependent on physical manifestations of the body. The participants become their disease (Dings & Glas, 2020; Hadler, 1996.) According to the accounts, the participant's spiritual needs are felt to be disconnected from the malfunctioning body. This detachment is underlined by the medical treatment, which is directed at physical recovery. Thereby, albeit unwillingly, both participants and doctors put the body-mind dualism into effect:

I didn't feel good in my body at all. From my 16^th^ they stuffed me with prednisone, which worked very fast, but because of it I gained a lot of weight in no time. Such a big face. And when I looked in the mirror I thought I did not feel the way I looked. That was very crazy. (P9)

You become a different person because of the disease. Everything turns around in your body, completely, also your feelings. You become kind of selfish, because you are in so much pain. (P11)

Becoming the embodiment of your own disease is often a devastating process for the participants. But their ability to survive is tested even more after their recovery. The participants have to reinvent themselves, which paradoxically is experienced as a burden. They have to leave the safe cocoon, as one participant puts it, where she had total control:

You have been outside the society for 14 years and it was quite a job to come back. I thought getting ill was a job. I believe it took me three years to surrender to it. But recovering took time as well. Because you had your own world, but now people expect you to be there. …I felt overwhelmed... (P2)

You’re 46, like a beautiful dead bird, you can’t do anything anymore. Eventually you climb up again a little bit. But you can do about 30 percent of what you did before. 24 years go by, and you are 70 [recovery date]. And then you must find out who you are. At 70, that’s a tough matter. (P1)

All participants are happy with their recoveries, but physically and mentally, they need time to extend their everyday life space beyond the restrictions imposed by their medical conditions. Their experiences, to use Bury’s terminology, can be seen as a second biographical disruption, or as a first step towards restoration of one’s wholeness. The participants feel the need to overcome uncertainty, to participate again. But this also means to be open to all kinds of reactions from their surroundings, including suspicion, distrust, jealousy and direct accusations of being a fraud or being a conduit of the devil’s work:

I had a lot of doubts about what was being said, am I healed, am I an impostor? ‘Was it not all in my mind?’ Because I heard that too: it was just in your head. I found that so difficult. Because how can I prove it? …Now I’ve learned, I don’t have to explain. People can believe it or not. My family believes it. I believe it. (P10)

Negative reactions to the recoveries within the church communities, families and among friends are mentioned in each of the interviews. The participants have to cope with an overwhelming number of questions, including self-doubts. Unsurprisingly, three of them end up with a burnout soon after their recovery, and at least two of them make use of psychological help and support.

Still, the positive transformative power of recovery appears to outweigh its challenges. More things are healed than the debilitating physical conditions, for instance the doubt whether the participant is worthy to be in this world, to be God’s child. Before the recovery some of them felt that they were not allowed to belong or to be special, like the participant who had been intimidated by his parents during his entire life because he was born as a boy and not a girl as they had expected, or like another participant, who questions her right to accept the healing: ‘I was standing onstage completely petrified, afraid that [disease] would come back, that I’m not good enough’. (P13).

This last participant learns to harness her uncertainty by straightening out her relationship with her mother, for whom she has become a social worker. Another participant takes the difficult decision not to see her sister anymore, because she feels she is being used by her. Another one speaks openly for the first time about things that felt wrong in her parental home, which gives her a feeling ‘as if the sky was falling’. The participants seem to experience the healing as just a first step in their pilgrimage towards feeling whole again.

The participants give a meaning to their recovery in relation to their life goals and future work, which leads to restoration of their selves that were temporarily lost to the illnesses. The pattern that emerges from the analysis comes close to a holistic outlook on life. The post-anorexia participant feels that her ‘mind and body have completely reunited’. All the participants maintain that their physical condition will remain stable, and that they are now concentrating on a more profound transformation after ‘being touched in your head or in your heart’ (P9):

God goes a little deeper. …It’s not just a physical healing, but it touches the soul. It is a relationship. This is not a doctor who does an operation. God gets really close. I think there is a lot more to heal within me too. (P13)

All the participants feel strengthened in their faith after their recovery. The majority feel a more profound connection with the world than before, which transcends the materiality of their existence. They give testimonies about their healing both within and outside church communities. Many have published their testimonies on the web or have written books about their experiences. When asked for clarification about their recovery, our participants react differently. Some of them are still looking for answers: ‘I still notice that this is the only thing I feel lonely about, because I’m so happy, but I have so many questions!’ (P6). Others simply feel content:

I am not interested in explanations. I’ve stopped trying to find any. Healing comes from God, because I don’t know anybody else who could do it. (P8)

## Discussion

The article presents the analysis of 14 cases of medically remarkable healings after prayer. Two aspects unite these cases and at least one distinguishes them from the studies on MUS or the placebo effect. Firstly, our cases follow a non-medical intervention, which sets them apart from recoveries within a clinical context. Secondly, the recoveries have a transformative power on various aspects of the participants’ lives, including their spiritual development. This second aspect unites our cases with other types of recoveries, like spontaneous remissions, which have been described elsewhere (Radin, 2021).

Since their remarkable recoveries, most of our participants chose to become engaged with their social environment: they do community (voluntary) work and use their own experiences in order to help others. They also engage in conversations about the transformative power of their recoveries (Levin & Steele, 2005), by making the accounts about their recoveries public (see e.g. Doodkorte, 2016). Most of them see it as their calling to spread the word about the extraordinary experiences they have gone through, whereas others are still searching for answers to questions like ‘why me?’ and ‘how can I share this gift with others?’.

To do justice to these complex processes, we developed a study design, based on several frameworks, including grounded theory and horizontal epistemology. By doing so we remained as closely as possible to the accounts of the respondents about their medical conditions, but also broadened the interpretative framework stepwise, by subsequently adding new perspectives: a biographical perspective, including the histories of spiritual development and the role of the life-events that shaped the patients’ views on life; a self-experiential perspective, with detailed descriptions of the healing, focusing on emotions and bodily sensations; and a spiritual perspective, including the patients’ personal views about God and their effect on their faith. A juxtaposition of the perspectives can be productive, even when they do not line up. This reaches the surface in the divergent reactions to the remarkable recoveries, including the reactions from the participants themselves. Doubt and confusion that the patients and doctors express, resonate with some of the responses within the church communities to which our patients belong, and also among their friends and families. Disbelief, suspicion or even jealousy of people who prayed but did not heal, emphasise the limitations of the cause-and-effect logic, which, in the Western cultural climate of naturalism offers little room for the unexpected (Jüngel, 1977).

Our framework, which contains medical, life-course and spiritual-quest discourses, emerges empirically and points at uncertainty as an important issue in both medical and spiritual reactions to HP. In the method-section we referred to inductive and abductive types of reasoning that we had used in order to understand unexpected and sometimes surprising examples in our data, like the temporal coincidence between recovery and HP. Abductive logic provided us with an opportunity to question existing (for example psychosomatic) explanations, and, while using logical inference, remain open for an unexpected insight (Reichertz, 2019).

Both doctors and patients are uncertain about how to deal with a remarkable recovery and how to integrate the discourse of spiritual development into the history of illness and recovery. Uncertainty does not fit well within the prevailing medical epistemology (Miles, 2009). This is somewhat surprising, because, as Fox points out, uncertainty is inherent in medical research and practice (2002); it has been present in the medical-sociological discourse since the work of Parsons (1951) and has been described in-depth in a number of publications (see e.g. Fox, 2002 on epistemological uncertainty and critique of evidence-based medicine; Han et al., 2021).

Scientific and technological progress in medical sciences has not eliminated the uncertainties within the available knowledge and explanations, but rather is making them more complex (Fox, 2002). That is why the patients may be left looking for their own sources of explanation, where medical explanation comes up short. The persons who have experienced a spiritual journey may well frame it in terms of a miracle (unexplained but positive). They can illustrate the trans-somatic aspect of their healing by showing the transformative effect that HP has had on their spiritual development and how a new transcendent dimension has been added to their lives. For the patients, healing is much more than a repair of a bodily function. It underscores the necessity of what Miles calls medicine for the whole person, which implies that disease is just a partial aspect with respect to a person, and that not everything that ‘…is right to the disease is automatically right for the patient’ (2009, p 944). In order to cover the full complexity of HP, follow-up studies are required, where cohesion of the physical, mental and spiritual aspects of recovery can be elucidated with the help of theological and philosophical theoretical perspectives.

This study has practical and academic implications. Firstly, we should look critically at the interaction between the patients and medical professionals, the persistent asymmetry of which has already been addressed in literature (Pilnick & Dingwall, 2011). Insight in the medical discourse can bring the patient closer to the medical specialist and ensure that they are on the same page where disease and treatment are concerned. But when unexpected healing takes place, confusion tends to take over. Our data suggest that in modern Western medicine we are hardly able to get a grip on such experiences of recovery. This can lead to self-suppressive and self-stigmatising behaviour on the part of the patients, with corresponding consequences for their mental and physical health (Charmaz, 2000).

There is no literature known to us about the language that is used during medical consultations where HP is discussed. We do see some similarities in the psychiatric literature regarding spiritual dimensions (Glas, 2021) and in the research on explanations that are used by doctors during consultations on MUS (Ring et al., 2005). Some authors focus on the psychosocial dimension of the disease (Stortenbeker et al., 2022). However, many patients feel offended by the association of their ailment with psychosomatic disorders. As Greco explains, labels such as ‘symptoms all in the mind’ touch on ‘moral failure’ and can ‘imply that the illness is imaginary, fake or inauthentic, possibly even intentional’ (2019, p. 104). The discourse surrounding HP touches on existential matters of life and therefore can be similarly ambiguous, and yet, based on our analysis, we advocate for making it part of medical consultation.

Secondly, the literature about the positive influence of spirituality and beliefs on health is abundant, but often overlooked in the Western medical literature reviews (Levin, 2020). The benefits of spiritual beliefs about health are therefore often wasted where medical treatment is concerned (Balboni & Peteet, 2017). Our analysis points out the importance of a multi-layered approach to the patient’s history, whereby the medical history forms only a part of the entire picture.

It is a challenge to implement that kind of approach, because patient-centred care and the efficiency of care ask for more and for less time respectively. Patient-centred care (Epstein, 1995) has brought along opportunities and tensions at the same time. Greco (2017) presents an analysis of those tensions, raising amongst others the important question of accountability. Following Stengers (2008), Greco advocates ‘creative accountability’ which, given our analysis, we can translate into being open to tentative or provisional and therefore uncertain forms of explanations. In that way the explanatory framework for the cases of remarkable recovery can be presented as a process of co-creation, where patients, doctors and possibly other stakeholders together are in search of an inventive understanding of a recovery (Glas, 2019; Savransky, 2017).

Finally, we have demonstrated that horizontal epistemology offers a fruitful approach to study HP. Horizontal epistemology departs from the assumption that there is no clear hierarchy or meta-theory to demonstrate why some types of knowledge matter more than others to understand a phenomenon (Abma, 2020). Horizontal epistemology is contrary to vertical epistemology, in which it is assumed that certain types of knowledge are more true than others. Yet, it is impossible to prove this convincingly, because there is no meta-theory that can be used.

So far most research on HP is grounded in a vertical epistemology. As a result, studies favour medical evidence over patient experiences and over psychological, sociological and theological interpretations of HP. The benefit of horizontal epistemology is that different explanations as well as frictions between epistemic discourses, are welcomed and can form a starting point for learning. This has offered new insights in how patients use and appropriate various discourses, to cope with an unexplained healing and how this can lead to tensions with people around them as well as with medical doctors. Also, it has enlarged and deepened our understanding of HP and offered a starting point for dialogue and deliberation across epistemic discourses, also within our project’s medical assessment team. We recommend that future studies of HP will be grounded in horizontal epistemology.

## Limitations

This study has several limitations. We focused on cases of recovery related to Christian prayer only. This decision was made intentionally, in order to keep a clear focus on the subject at hand. It prevents us however from comparing experiences of people with different beliefs and of non-religious people. Furthermore, we are aware that our interpretations only mirror attitudes that are existing in the Western cultures, where all the members of the research team are living and working. Finally, due to our limited time and resources, we have interviewed former patients only. Their medical specialists were contacted with requests to provide the medical files only. It would be worthwhile to gather first-hand data from medical specialists about their experiences with remarkable recoveries and HP, in order to fully enact horizontal epistemology. Church members, friends and family members of the participants were not interviewed, which limits our understanding of the context within which our participants live.

## Conclusion

Summarising, our analysis of the data allows us to see that in the effort to understand cases of remarkable recovery, we require a combination of discourses and interpretative frameworks that include uncertainty as a means of (not-)knowing. Each of the discourses and frameworks has its value and none of them can be sufficient on its own. In order to understand the cases better, transdisciplinary analysis is required, where various discourses challenge each other in a process of co-creation. Allowing uncertainty of the unknown into a consultation, confession or interview, can boost the inventive side of our ability to understand and explain these cases.
